# Tackling HIV in MENA: Talk Is Not Enough–It Is Time for Bold Actions: A Response to Recent Commentaries

**DOI:** 10.15171/ijhpm.2017.110

**Published:** 2017-09-10

**Authors:** Mohammad Karamouzian, Navid Madani, Fardad Doroudi, Ali Akbar Haghdoost

**Affiliations:** ^1^HIV/STI Surveillance Research Center, and WHO Collaborating Center for HIV Surveillance, Institute for Futures Studies in Health, Kerman University of Medical Sciences, Kerman, Iran.; ^2^School of Population and Public Health, University of British Columbia, Vancouver, BC, Canada.; ^3^Department of Cancer Immunology and Virology, Dana-Farber Cancer Institute, Department of Global Health and Social Medicine, Harvard Medical School, Boston, MA, USA.; ^4^UNAIDS – The Joint United Nations Programme on HIV/AIDS (UNAIDS), Tehran, Iran.

We appreciate the insightful and thought-provoking commentaries by Kaplan and El Khoury^1^ and Modjarrad and Vermund^2^ about our recent editorial entitled “Improving the quality and quantity of HIV data in the Middle East and North Africa: key challenges and ways forward.”^3^ Kaplan and El Khoury highlight the challenges faced by lesbian, gay, bisexual, and transgender (LGBT) communities and offer insight in improving HIV surveillance efforts among this marginalized population in the conservative context of the Middle East and North Africa (MENA).^1^ Modjarrad and Vermund explain how the existing public health infrastructures and data systems could enhance the quality and quantity of HIV-related data in the region and highlight the need for intensifying the “follow-up, care and data collection efforts for known HIV cases.” They also discuss some real world examples about reaching key populations effectively despite their ‘criminalized’ status.^2^ We completely agree with their excellent suggestions about taking full advantage of network of existing “surveillance, clinical and research data repositories, along with information-sharing agreements across the MENA region.”^2^ However, considering the slowly evolving nature of the epidemic in the region, MENA countries need to invest further in learning from the global experience and optimizing the intervention outputs in order to evade the future burden of HIV on their population and healthcare system.

We also agree about the importance and feasibility of reaching key populations and welcome the thoughtful suggestions for improving LGBT engagement in HIV surveillance efforts.^1,2^ Furthermore, we would like to highlight the importance of modifying the language around key populations at risk of HIV across MENA as a useful strategy in linking them to prevention and care services. For example, the inclusion of female sex workers (FSWs) in Iran’s HIV surveillance programs, despite their ‘criminalized’ status in the country’s Judiciary system, was largely facilitated by changing the former lexicon around FSWs (ie, ‘street women’ or ‘street prostitutes’) to the culturally and religiously sensitive term of ‘vulnerable women’ (ie, women who need help).^4^ Subsequently, Iranian FSWs are now provided with a number of sexual- and drug-related services through “Centres for Vulnerable Women” which have been successfully expanded; there are now over 35 centers across the country that continue to offer services to FSWs, women who use/inject drugs, homeless women, and female sexual-partners of at-risk individuals.^3,5-8^ Similar strategies could be explored across other MENA countries where concerns exist around supporting ‘criminalized’ populations including FSWs and men who have sex with men.

On the other hand, one size will not fit all and any public health intervention needs to be culturally, ethnically, and religiously sensitive in order to be beneficial in providing key populations with services without crossing the ‘red-lines’ of the ruling governments in Muslim-majority MENA countries. Considering that “MENA countries comprise a complex mosaic of populations and policies,”^2^ uptaking a country-specific causal layered analysis approach – a method that goes beyond the conventional framing of the problem and examines all of the deep and shallow influential factors based on their level of effect (ie, litany, causes, worldview, and metaphor levels) – could be a game changer in addressing the HIV epidemic in MENA in the near future.^9-12^

Lastly, we would like to acknowledge that the recommendations that we and other scholars have made about addressing the HIV epidemic in MENA are of limited value if they are not put into action. We are really keen to see further empirical high quality HIV research coming out of MENA to provide a better picture of the HIV epidemic in the region. We believe that getting close to UNAIDS 90-90-90 targets requires further work across the MENA region and it is time for taking bold actions.

## Ethical issues

Not applicable.

## Competing interests

Authors declare that they have no competing interests.

## Authors’ contributions

All authors contributed to the conceptualization of the correspondence. MK drafted the correspondence and all authors reviewed and aproved the final draft.

## Authors’ affiliations

^1^HIV/STI Surveillance Research Center, and WHO Collaborating Center for HIV Surveillance, Institute for Futures Studies in Health, Kerman University of Medical Sciences, Kerman, Iran. ^2^School of Population and Public Health, University of British Columbia, Vancouver, BC, Canada. ^3^Department of Cancer Immunology and Virology, Dana-Farber Cancer Institute, Department of Global Health and Social Medicine, Harvard Medical School, Boston, MA, USA. ^4^UNAIDS – The Joint United Nations Programme on HIV/AIDS (UNAIDS), Tehran, Iran.
